# Toward an Integrated Genome-Based Surveillance of *Salmonella enterica* in Germany

**DOI:** 10.3389/fmicb.2021.626941

**Published:** 2021-02-10

**Authors:** Laura Uelze, Natalie Becker, Maria Borowiak, Ulrich Busch, Alexandra Dangel, Carlus Deneke, Jennie Fischer, Antje Flieger, Sabrina Hepner, Ingrid Huber, Ulrich Methner, Jörg Linde, Michael Pietsch, Sandra Simon, Andreas Sing, Simon H. Tausch, Istvan Szabo, Burkhard Malorny

**Affiliations:** ^1^Department of Biological Safety, German Federal Institute for Risk Assessment, Berlin, Germany; ^2^Department of Food, Feed and Commodities, Federal Office of Consumer Protection and Food Safety, Berlin, Germany; ^3^Bavarian Health and Food Safety Authority, Oberschleißheim, Germany; ^4^Unit of Enteropathogenic Bacteria and Legionella (FG11) – National Reference Centre for Salmonella and Other Bacterial Enteric Pathogens, Robert Koch Institute, Wernigerode, Germany; ^5^Institute of Bacterial Infections and Zoonoses, Friedrich-Loeffler-Institut, Jena, Germany

**Keywords:** *Salmonella*, surveillance, food-borne disease outbreak, whole genome sequencing, cgMLST

## Abstract

Despite extensive monitoring programs and preventative measures, *Salmonella* spp. continue to cause tens of thousands human infections per year, as well as many regional and international food-borne outbreaks, that are of great importance for public health and cause significant socio-economic costs. In Germany, salmonellosis is the second most common cause of bacterial diarrhea in humans and is associated with high hospitalization rates. Whole-genome sequencing (WGS) combined with data analysis is a high throughput technology with an unprecedented discriminatory power, which is particularly well suited for targeted pathogen monitoring, rapid cluster detection and assignment of possible infection sources. However, an effective implementation of WGS methods for large-scale microbial pathogen detection and surveillance has been hampered by the lack of standardized methods, uniform quality criteria and strategies for data sharing, all of which are essential for a successful interpretation of sequencing data from different sources. To overcome these challenges, the national GenoSalmSurv project aims to establish a working model for an integrated genome-based surveillance system of *Salmonella* spp. in Germany, based on a decentralized data analysis. Backbone of the model is the harmonization of laboratory procedures and sequencing protocols, the implementation of open-source bioinformatics tools for data analysis at each institution and the establishment of routine practices for cross-sectoral data sharing for a uniform result interpretation. With this model, we present a working solution for cross-sector interpretation of sequencing data from different sources (such as human, veterinarian, food, feed and environmental) and outline how a decentralized data analysis can contribute to a uniform cluster detection and facilitate outbreak investigations.

## Introduction

The surveillance of zoonotic pathogens is an important task usually conducted by official authorities. The early detection of food-borne pathogens has a crucial role in reducing the major clinical and economic burden caused by food-borne disease outbreaks (Jain et al., 2019). Whole-genome sequencing (WGS), combined with data analysis, is a high throughput technology with an unprecedented discriminatory power, which is increasingly used for cluster detection, source tracking, outbreak investigation and surveillance. WGS data are highly informative, portable and standardizable (Deng et al., 2016). Although WGS is recognized as the most up-to-date methodology for the detection of infection clusters and its use is highly encouraged by international authorities (ECDC, 2016), efficient real-time surveillance using WGS requires the development and implementation of a functional cross-sectional concept (covering public health, veterinarian, food, feed and environmental sectors). A successful concept should be designed to prospectively detect clusters of very similar isolates based on the WGS data and thus requires the use of powerful bioinformatics tools. The combination of sequence data analysis with relevant metadata and commodity chain information allows to trace transmission paths and to identify possible sources of outbreaks, thereby improving consumer protection and microbial food safety (Aarestrup et al., 2012; Moura et al., 2017; EFSA Panel on Biological Hazards, 2019).

Up to date, genome-based surveillance in most European countries, if implemented, is mainly realized through stand-alone solutions. As a consequence, different quality parameters and analysis processes are employed in different laboratories. This represents a major challenge for a cross-sectoral consolidation of data, which is needed for a consistent interpretation of results. Without a uniform quality of data sets and subsequent interpretation of data, it is not possible to reliably identify coherencies, assess risks and take measures to contain outbreaks and emerging epidemic clones.

A number of countries have established, or are currently establishing national WGS-based surveillance systems and platforms, such as a the United Kingdom (Public Health of England) (Ashton et al., 2016) and Switzerland (Egli et al., 2018). At the same time, large international platforms, such as Pathogenwatch^1^, INNUENDO (Llarena et al., 2018), NCBI pathogen detection^2^ and GenomeTrakr (Allard et al., 2016; Timme et al., 2018) have been developed to enable the analysis of multinational genome sequence data for the surveillance and investigation of cross-border transmissions and outbreaks.

All aforementioned platforms have in common that they require raw sequencing data (and often accompanying metadata) to be deposited in a central database in order to facilitate a centralized analysis of the genome data. Although an enhanced surveillance system would ideally encompass genome data on a global scale, the requirement for centralized data storage, combined with the existence of specific legislative responsibilities at local, regional or federal level, in practice often prevents or delays the participation of countries or institutions in these networks. Overall, there is no one-fits-all solution which can be easily adapted and which does not require central data storage and sharing.

As a consequence, in countries where sequence data is not shared in real-time between sectors, public health and food control laboratories, the potential of sequencing data is not fully exploited for cluster detection.

Here, we present the ‘GenoSalmSurv’ project (‘Integrated genome-based surveillance of *Salmonella’*), which targets these challenges and develops a practical approach for the genome-based cross-sectoral surveillance of the food-borne pathogen *Salmonella*. The project is funded by the German Federal Ministry of Health and part of a funding framework on integrated genome-based surveillance of zoonotic pathogens. The project is carried out by the Robert Koch Institute (RKI) hosting the National Reference Centre for *Salmonella* and other Bacterial Enteric Pathogens (NRC-*Salmonella*) (human sector) and the German Federal Institute for Risk Assessment (BfR) hosting the NRL-*Salmonella* (food, feed, animal, environmental sector), as well as the Bavarian Health and Food Safety Authority (LGL) as representative state authority (regional animal, food and human sector). Two further partners are associated, the ‘NRL for Salmonellosis in Cattle’ at the Friedrich-Loeffler-Institut (FLI) (epizootic disease sector) and the federal contact point for food-borne outbreaks, which is located at the Federal Office of Consumer Protection and Food Safety (BVL). The main goals of the project are (i) to establish standard procedures for the joint use of the high-throughput WGS data of *Salmonella* spp. from different sectors, collected from monitoring programs and surveillance/sentinel programs, and (ii) to supply open-source tools for the subsequent bioinformatics analysis, which enables strain comparison on a high resolution level. Two major challenges are thus addressed: On the one hand the establishment of laboratory parameters for WGS and on the other hand the development and harmonization of bioinformatics procedures. These involve all steps from genome assembly (including read and assembly QC), to *in silico* serotyping and automated cluster detection. Selected tools for the bioinformatics procedures are intended to be internationally recognized and scientifically validated and will be additionally evaluated for applicability. A particular focus is on the open-source availability of the selected tools to provide wide accessibility and sustainability.

To ensure the practicability of the developed processes and protocols, the project conducts a proof-of-principle study (real-time application phase). During this 6-months period, about 2,300 *Salmonella* spp. isolates of human, animal and food origin, assigned to conventional typing, are additionally analyzed according to the workflow established in the GenoSalmSurv project. The participating partners (RKI, BfR, LGL) apply the developed procedures with the aim to detect emerging clusters and to identify relevant surveillance markers. To ensure a manageable scope, the focus is set on the serovars *Salmonella enterica* subsp. *enterica* (herein after abbreviated as *S.*) Enteritidis, *S.* Typhimurium and *S.* Infantis, which are not only responsible for most infections (70–80%) and outbreaks in humans (RKI, 2015, 2016, 2017), but also represent the most prevalent serovars in certain production animal species. Further, additional serovars of interest are included [e.g., *S.* Choleraesuis, known as swine-adapted serovar, but highly systemic in humans (Chiu et al., 2004)]. The insights gained from this proof-of-principle study will be evaluated in terms of quality control, user friendliness and data sharing strategies. However, this study might reveal further needs for improvements, covering prospective outbreak detection (e.g., effects on defining priorities and responsibilities, communication and potential increased risk management needs).

Here, we elucidate the past and current epidemiology in animal, food and humans, as well as important outbreaks of *Salmonella* in Germany. We present a working model (‘GenoSalmSurv’) for the establishment of a *Salmonella* surveillance, as well as source tracking system based on WGS data. Our approach for an integrated genome-based surveillance aims to strengthen the collaboration of public health and food safety authorities and allows authorities to rapidly detect *Salmonella* food-associated disease clusters, which supports a more focused epidemiological outbreak investigation.

## Materials and Methods

### Reporting *Salmonella* Epidemiology and Outbreaks in Germany

**FIGURE 1:**
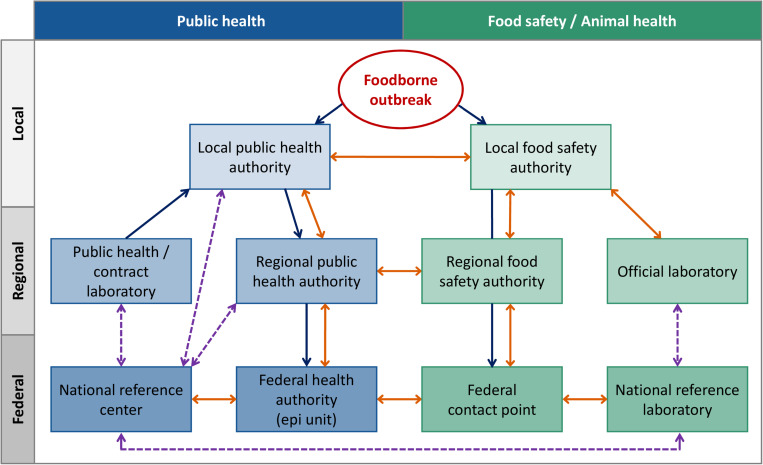
Overview on reporting (blue), information exchange (orange), and subtyping activities (dashed line, purple) for food-borne outbreaks between the German public and animal health as well as food safety sector.

The German Protection against Infection Act (IfSG)^3^ lays down the notifiable diseases and mandatory deadlines for reporting. The obligation to notify includes the suspicion of and disease from microbial food poisoning or acute infectious gastroenteritis, if two or more similar diseases occur for which an epidemic link is probable or suspected (§6 IfSG). Further, any direct or indirect evidence of certain pathogens (including *Salmonella* spp.) in patients shall be notified if indicating an acute infection (§7 IfSG). According to these legal requirements, public health laboratories or practitioners report to the local health authorities who evaluate the notifications and transmit the anonymized data to the state health departments. In 2001, the electronic reporting system for surveillance of notifiable infectious diseases [SurvNet@RKI (Faensen et al., 2006)] was established. Notifications from the regional bodies are finally recorded and analyzed at the RKI, the German federal public health authority. Food-borne outbreaks can also be identified by consumer complaints to the food safety authorities (Figure 1). According to the Protection against Infection Act and the German Food, Feed and Consumer Goods Code^4^, there is a mutual information obligation between the public health and food safety sector on local level. Germany is a federal state where the competence for food-borne outbreak investigations lies with the local authorities. In the case of outbreaks spreading across jurisdictions, superior federal state authorities and federal authorities can be involved. The flow of information is depending on federal state structures and emergency contingency plans of the federal states. Cooperation between laboratories is necessary for subtyping of isolates, as well as comparison of typing data between the sectors. Depending on laboratory capacities in the 16 federal states, isolates for outbreak investigations are subtyped on regional or federal level. Public health laboratories voluntarily forward human isolates for typing to the NRC-*Salmonella* at the RKI. National Reference Centers are appointed for a 3-year period by the Federal Ministry of Health as competence centers in the field of laboratory science for a particular pathogen or group of pathogens. There is a regular evaluation by the scientific advisory board for public health microbiology and expert reviewers. Official food laboratories send isolates from food, production environments and primary production to the NRL-*Salmonella* at the BfR. In addition, the ‘NRL-for *Salmonella* in Cattle’ at the FLI investigates *Salmonella* isolates obtained from cattle in Germany. Both NRLs perform their work in accordance with the Zoonoses Directive 2003/99/EC^5^, the Zoonoses Regulation (EC) No 2160/2003^6^, and the §64 of the German Food, Feed and Consumer Goods Code. The typing results are communicated downstream within sectors (Figure 1). If there is an outbreak affecting more than one federal state, epidemiological information and concise typing data are shared between the sectors *via* the federal contact point for food-borne outbreaks, which is located at BVL.

### WGS Tools Selected for the Working Model for the Surveillance of *Salmonella* spp.

**FIGURE 2:**
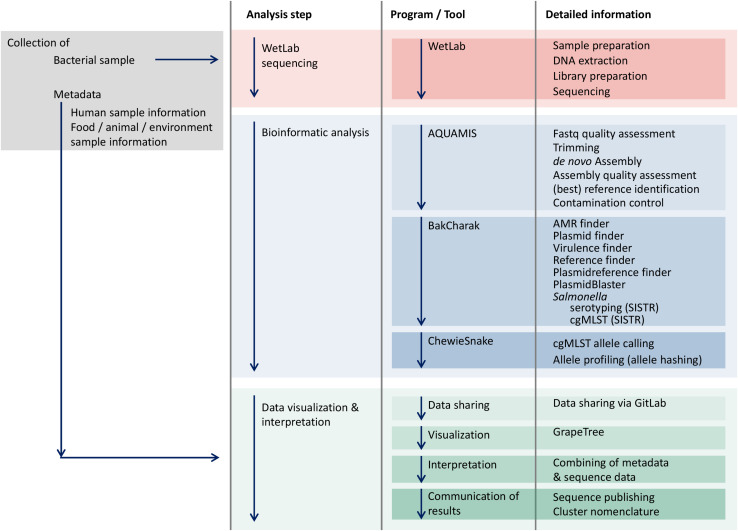
Workflow of the GenoSalmSurv working model.

For the decentralized initial bioinformatics analysis of raw sequencing data on site, a number of freely available, open-source pipelines have been customized for performing (i) quality control and assembly, (ii) bacterial characterization, (iii) cgMLST-based subtyping and (iv) phylogenetic analysis (Figure 2). The AQUAMIS pipeline (Assembly-based QUality Assessment for Microbial Isolate Sequencing)^7^ encompasses trimming and read-quality control (QC) using fastp (Chen et al., 2018), assembly using shovill^8^, automated reference search of complete NCBI Refseq genomes using mash (Ondov et al., 2016), and assembly-quality analysis using Quast^9^. Based on the draft assemblies produced by AQUAMIS, the Bakcharak pipeline^10^ conducts a characterization of the *Salmonella* genome. The Bakcharak pipeline runs NCBI amrfinder (Feldgarden et al., 2019) to detect antimicrobial resistance genes and ABRicate^11^ to detect plasmid incompatibility groups from the plasmidfinder database (Carattoli et al., 2014), as well as virulence genes from the VFDB (Chen et al., 2004). Moreover, 7-gene MLST is performed using mlst^12^ and the PUBMLST schemes (Jolley and Maiden, 2010). The *Salmonella* serovar is determined with the SISTR software (Yoshida et al., 2016). The chewieSnake pipeline^13^ performs cgMLST typing by implementing the assembly-based software chewBBACA (Silva et al., 2018). The alleles for a set of samples are called using chewBBACA v2.0.12, allele profiles are then combined and an allele distance matrix and minimum spanning tree are calculated using GrapeTree v2.1 (Zhou et al., 2018). The cgMLST scheme for *Salmonella* was derived from Enterobase^14^. The AQUAMIS, Bakcharak and chewieSnake bioinformatic pipelines are version controlled and associated with release tags. All contained software versions are fully tracked with the conda software management tool.

## Results

### Epidemiology of *Salmonella* in Germany

Salmonellosis is one of the most common bacterial food-borne illnesses worldwide, with approximately 153 million cases and 57,000 deaths per year (Healy and Bruce, 2017). Despite the remarkable and steady drop of reported human *Salmonella* infections from almost 195,000 in 1992 to about 14,000 in recent years, salmonellosis is, after campylobacteriosis, still the second most frequent bacterial food-borne disease in Germany. In the 5-year period from 2015 to 2019 (RKI, 2016, 2017, 2018, 2019) the number of reported cases ranged from 12,962 to 14,269, representing an incidence of 16 to 17 cases per 100,000 inhabitants. The highest incidence has always been found in children younger than 5 years. The majority of human salmonellosis cases in Germany develops gastrointestinal symptoms, such as (generally self-limiting) diarrhea and stomach cramps that can be accompanied by fever, nausea or headache. Blood stream infections predominantly occur in elderly and immunocompromised individuals. Despite a relatively high median hospitalization rate (39%), only 14 to 20 fatalities (with salmonellosis stated explicitly as cause of death) had been reported per year from 2015 to 2019. For decades, the predominant serovars causing human infections in Germany have been *S.* Typhimurium and *S.* Enteritidis. Together, they regularly account for almost 80% of all cases^15^ with reported serovars in Germany (RKI, 2016, 2017, 2018, 2019). However, for a considerable number of cases, the organism is reported only on serogroup (O-antigen), but not on serovar (combination of *O*- and two *H*-antigens) level, so that the actual numbers of cases with these two serovars may even be higher. Far behind, the next ranked reported serovars (each <3% of reported cases) have been *S.* Infantis and *S.* Derby.

The prevalence of *Salmonella* in animals and food products is rather low in Germany. According to the German National *Salmonella* Control Program in 2019 the prevalence of *Salmonella* in poultry for any of the five target serovars (*S.* Enteritidis, *S.* Typhimurium, *S.* Virchow, *S.* Infantis and *S.* Hadar) was less than 1% in breeding flocks, broilers and fattening turkeys, and 1.1% in laying hens and breeding turkeys. The dominating serovars in poultry were *S.* Enteritidis and *S*. Typhimurium (BFR, 2020).

In Germany, outbreaks of salmonellosis in cattle herds, officially confirmed by the competent authority, are notifiable. Between 2010 and 2019, approximately 110–130 outbreaks of bovine salmonellosis were recorded each year^16^. *S.* Typhimurium was the dominating serovar and caused 40 to 50% of the annually reported outbreaks. The host-adapted serovar *S.* Dublin amounted to 30 to 40% and *S.* Enteritidis caused about 5 to 10% of all registered outbreaks. Approximately 15 to 20% of outbreaks were allocated to other serovars, with no single serovar in that group, showing an increasing detection rate over longer times (Methner, 2019).

In 2015 and 2016 within the frame of the National Zoonosis Monitoring Program, fecal samples from pigs were tested and detected as *Salmonella* positive in 5.6% of breading sows, 10.3% of runners (BVL, 2016a,b) and 7.9% of fattening pigs (BVL, 2018a,b). The most prevalent serovars in fattening pigs were *S.* Typhimurium and its monophasic variant, whereas the most frequent serovars in sows were *S.* Derby followed by *S.* Typhimurium.

Within the National Zoonosis Monitoring Program, *Salmonella* was detected less frequently in fresh pork and beef than in fresh poultry meat in Germany. The dominating serovars in poultry products were *S.* Infantis, followed by *S.* Paratyphi B d-tartrate fermenting (*dT*+), and *S.* Typhimurium, as well as its monophasic variant in pork (BVL, 2018a, 2019). Considering foods of plant origin, the NRL-*Salmonella* only sporadically receives isolates for further serotyping, indicating a rather low prevalence. This is confirmed by the EU-wide monitoring data which revealed a *Salmonella* detection rate of only 0.84% in ready to eat pre-cut fruits and vegetables (EFSA and ECDC, 2017), although outbreaks were detected (Table 1).

**TABLE 1:** Selected *Salmonella* outbreaks in Germany since 2000.

Year	Serovar	Cases	Suspected vehicle of infection	Remarks	References
2018/2019	*S.* Enteritidis	>300	Eggs, egg products		Unpublished
2018	*S.* Enteritidis	30	‘Spätzle’ (fresh pasta with egg)		RKI (2019)
2018	*S.* Panama	28	Not identified	Retirement home	RKI (2019)
2017	*S.* Enteritidis	52	Not identified	Day-care center for children	RKI (2018)
2017	*S.* Kottbus	50	Ham	At the same time a smaller *S.* Kottbus outbreak related to quail eggs	Enkelmann et al. (2020)
2017	*S.* Typhimurium	31	Pork products		RKI (2018)
2017	*S.* Enteritidis	33	Bakery products		RKI (2018)
2016	*S.* Enteritidis	20	Not identified	Retirement home	RKI (2017)
2016/2017	*S.* Vari	47	Sesame products	Multinational, 13 German cases; novel serovar	Meinen et al. (2019)
2016	*S.* Enteritidis	14	Potato salad		RKI (2017)
2014	*S.* Muenchen	247	Pork products	Common source of 2013/2014 outbreaks in the primary production	Schielke et al. (2017)
2014	*S.* Enteritidis	>350	Eggs, egg products	Multinational, majority of cases in the United Kingdom	Dallman et al. (2016)
2014	*S.* Typhimurium	40	Ground pork		RKI (2015)
2014	*S.* Bovismorbificans	74	Sprouts	49 German cases, 25 in Switzerland	Knoblauch et al. (2015); RKI (2015)
2013/2014	*S.* Derby	145	Pork products (‘Teewurst’)	Hospitals and retirement homes	Simon et al. (2018)
2013	*S.* Muenchen	203	Pork products	Common source of 2013/2014 outbreaks in the primary production	Schielke et al. (2017)
2013	*S.* Infantis	267	Pork products		Schroeder et al. (2016)
2011	*S.* Strathcona	71	Tomatoes	Multinational, 21 German cases	Müller et al. (2016)
2011	*S.* Newport	106	Sprouts		Bayer et al. (2014)
2010	*S.* Montevideo	15	Food supplement	Hemp-based herbal food supplement	Stöcker et al. (2011)
2004	*S.* Give	115	Ground pork		Jansen et al. (2005)
2002/2003	*S.* Agona	42	Aniseed	Aniseed-fennel infusion for infants	Koch et al. (2005)
2001	*S.* Oranienburg	>400 (estimated)	Chocolate	Multinational	Werber et al. (2005)

Table 2 shows the top five *Salmonella* serovars in food (all categories), laying hens, broilers, pigs and cattle serotyped in 2019 at the NRL-*Salmonella* and the ‘NRL for Salmonellosis in Cattle’ in Germany. Isolates from laying hens, pigs and cattle were collected at primary production stage, while strains from food originate from all production stages.

**TABLE 2:** Top five *Salmonella* serovars from German food, laying hens, broilers, pigs, and cattle isolates submitted in 2019 to NRLs for serotyping.

Food (all categories)		Laying hens		Broilers		Pigs		Cattle	

*n* = 750		*n* = 143		*n* = 52		*n* = 995		*n* = 307	

Serovar	%	Serovar	%	Serovar	%	Serovar	%	Serovar	%
*S.* Typhimurium, monophasic	14.5	*S.* Enteritidis	43.8	*S.* Infantis	23.1	*S.* Typhimurium, monophasic	40.7	*S.* Typhimurium	36.4
*S.* Typhimurium	13.6	*S.* Typhimurium	13.7	*S.* Paratyphi B *dT*+	17.3	*S.* Typhimurium	18.8	*S.* Typhimurium, monophasic	13.2
*S.* Infantis	12.8	*S.* spp. I rough^1^	5.5	*S.* Enteritidis	15.4	*S.* Derby	18.3	*S.* Dublin	27.4
*S.* Derby	10.0	*S.* Mbandaka	4.8	*S.* Saintpaul	5.8	*S.* spp. I rough^1^	4.6	*S.* Enteritidis	7.1
*S.* spp. I rough^1^	8.5	*S.* Kiambu	2.7	*S.* Coeln	5.8	*S.* Infantis	2.1	other serovars	15.9

*^1^*S.* spp. I rough: *Salmonella enterica* subspecies *enterica* harboring a rough lipopolysaccharide disabling serovar determination.*

### Salmonellosis Outbreaks in Germany

*Salmonella enterica* regularly causes large (supra-)regional food-borne outbreaks in Germany (Table 1) and thus is a pathogen of extraordinary public health and economic relevance (EFSA and ECDC, 2018). In the 5-year period from 2014 to 2018, approximately 260 salmonellosis outbreaks had been reported per year, comprising a median of 1,090 cases/year. The number of cases per outbreak ranged from 2 to 191. Occasionally, local outbreaks have been detected in day-care centers for children or retirement homes.

A high number of outbreaks are caused by the most prevalent serovars *S.* Enteritidis and *S.* Typhimurium. *S.* Enteritidis has frequently been associated with eggs, egg products or egg dishes like Tiramisu and other (raw) egg-containing desserts, bakery products, ‘Spätzle’ (regional pasta-like specialty) or potato and pasta salads with mayonnaise. In contrast, *S.* Typhimurium outbreaks are often related to pork products, such as ground pork or different types of raw sausage (traditional meals in Germany). Both serovars might also be found in other food categories. These observations strongly reflect the situation in the EU (EFSA and ECDC, 2019).

In addition, food of plant origin like fruit and vegetables, herbs, spices and sprouts have been identified increasingly often as a source of *Salmonella* infections and outbreaks (Table 1). Foodstuffs contaminated with less frequent or even extremely rare serovars have also been associated with partly large (multinational) outbreaks.

In the summer of 2014, a multinational outbreak of *S.* Enteritidis was associated with an international egg distribution network (Dallman et al., 2016). The outbreak included more than 350 cases, reported in the United Kingdom, Germany, Austria, and Luxembourg. WGS investigations and phylogenetic analysis revealed a common ancestral relationship which indicated one particular German egg producer. The strength of the study was that WGS data in combination with information from the food supply network was applied. This approach enabled the trace-back to suspected sources across Europe categorizing the outbreak as multinational.

A few months earlier, one large salmonellosis outbreak, caused by *S.* Derby (a prevalent serovar in pigs), affected 145 primarily elderly people in hospitals and nursing homes (EFSA and ECDC, 2015; Simon et al., 2018) in the German states of Berlin and Brandenburg. Epidemiological investigations and additional microbiological evidence revealed raw fermented meat paste (“Teewurst”) as the source. This point source outbreak was retrospectively investigated by WGS with special attention on the suitability of SNP and cgMLST based methods for cluster definition (Simon et al., 2018). In total, 55 isolates were selected (confirmed outbreak strains, probable outbreak strains and unrelated ones) and analyzed. Overall, the WGS approach confirmed the conventional typing results and additionally identified two of the seven probable outbreak strains as part of the outbreak.

Over a period of 2 years, during 2016 and 2017, a previously undescribed *Salmonella* serotype 11:z41:e,n,z15, now defined as *S.* Vari, caused a multi-national salmonellosis outbreak. The first cases were reported in Greece (Mandilara et al., 2016), followed by cases in Germany, Czechia, Luxembourg, France, Serbia and the United Kingdom. Using WGS, a very close genetic relationship of isolates from different countries was observed and further epidemiological analysis revealed sesame products, processed in a Greek factory, as vehicles of infection. Samples from raw material and processed food acquired in the course of the event, tested positive for *S.* Vari and could be confidently attributed to human cases by WGS, taking into account the epidemiological data (Meinen et al., 2019).

In 2017, a multi-state outbreak of the rare serotype *S.* Kottbus in Germany involved 69 cases (Enkelmann et al., 2020). WGS enabled the differentiation of isolates belonging to the potential outbreak into three independent co-circulating clusters. For two of the identified clusters, the vehicle of transmission could be ascertained by epidemiological investigation: one was associated with raw smoked ham consumption, the other with quail egg consumption. For the third cluster no common food item was identified. Without WGS, attribution of all cases to just one event would have weakened the epidemiological evidence obtained from questionnaires and therefore impeded the identification of the vehicle of the first cluster outbreak and obscured the strong common exposure of quail egg consumption reported by the second cluster cases.

In 2017, a *S.* Agona outbreak among infants was observed, which involved 37 cases in France and two further international cases. Epidemiological and WGS investigation revealed infant milk products from a French supplier as the source (Jourdan-da Silva et al., 2018). Simultaneously, *S.* Agona was detected in three animal feed samples in Bavaria. In a retrospective WGS study, including the feed isolates and 48 additional *S.* Agona isolates (Bavarian isolates from 1993 to 2018), a connection between the feed isolates and the French outbreak was ruled out (Dangel et al., 2019). Furthermore, the WGS approach confirmed clusters that were previously identified by epidemiological investigations and detected additional ones.

### The GenoSalmSurv Working Model for Cluster Detection and Bioinformatics Analysis Tools Used for Real-Time Surveillance

Beside the generation of high quality sequencing data, data sharing between laboratories is a necessary requirement for efficient outbreak investigations (Aarestrup and Koopmans, 2016; FAO, 2016; Jagadeesan et al., 2019). However, any data sharing strategy needs to evolve around currently existing data protection regulations. Legal and ethical issues comprising confidentiality, data protection and intellectual property rights are of great concern (Pisani and AbouZahr, 2010; Roche et al., 2014). All parties involved in the handling of sequencing and metadata during outbreak and surveillance studies need to legally comply with such regulations. Previous studies have shown that many different interests hamper the sharing of data and these discrepancies still exist (Aarestrup and Koopmans, 2016; FAO, 2016). On European level, ECDC (European Centre for Disease Prevention and Control) and EFSA work closely together in outbreak detection and the EU legislation is considering the use of WGS data wherever an application is reasonable (EFSA Panel on Biological Hazards, 2019). The sharing of metadata along with genome sequence data, is a particular challenge as data protection is of high consideration in Germany. This restricts the exchange of sequence and metadata between federal states, as well as across sectors and thus complicates evaluation of outbreak analyses. To address these issues, joint working groups have begun to elaborate on WGS data sharing concepts. The aim is to reach a broad acceptance for data sharing embedded within a legal framework, in order to fully benefit from the application of WGS methodologies in food safety and public health.

Recognizing the existing barriers in data sharing, the GenoSalmSurv project has developed a practical technical solution for performing *Salmonella* real-time surveillance across all sectors. The model is based on a decentralized initial bioinformatics analysis of raw sequencing data on site, dispensing a direct exchange of raw sequence data between partners. Each partner utilizes their own local computing infrastructure and communicates results in a standardized format which facilitates data exchange (shrinking data exchange volume) and joint interpretation (harmonized results). A prerequisite for this approach is the use of harmonized analysis pipelines. Importantly, these pipelines must be easy to deploy, maintain and run on different (Linux) computers and servers. For the joint analysis, freely available, open-source pipelines have been customized for performing (i) quality control and assembly, (ii) bacterial characterization, (iii) cgMLST-based subtyping and (iv) phylogenetic analysis (Figure 2).

For the simplification of the local installation of all software components of the pipelines, the conda/bioconda software management tool is used, which ensures that every local system is equipped with the exact same software versions (Grüning et al., 2018).

For initial analyses of the bacterial isolate we deployed the AQUAMIS pipeline (Assembly-based QUality Assessment for Microbial Isolate Sequencing) (see text footnote 7). This snakemake pipeline (Koster and Rahmann, 2012) encompasses trimming and read-quality control (QC) using fastp (Chen et al., 2018), assembly using shovill (see text footnote 8), automated reference search of complete NCBI Refseq genomes using mash (Ondov et al., 2016), and assembly-quality analysis using Quast (see text footnote 9). The AQUAMIS pipeline produces a user-friendly html report, which summarizes essential quality information. Important quality parameters, like the Q30 base fraction and the coverage depth are color-coded according to the traffic light system (green – good, yellow – sufficient, red – insufficient), which facilitates an easy visual first-pass inspection. A number of other quality parameters are calculated for a subsequent in-depth analysis of samples of interest.

Table 3 summaries important minimal quality control parameter for WGS of *Salmonella* isolates based on our experience with library preparation using the Nextera DNA Flex library preparation protocol with subsequent sequencing on an Illumina platform (MiSeq, NextSeq or iSeq). Other examples for minimal quality parameters determined for sequencing of bacterial isolates were previously published by Kozyreva et al. (2017) (Nextera XT library preparation and MiSeq sequencing).

**TABLE 3:** Minimal quality parameters for Illumina sequencing of *Salmonella* isolates.

Parameter	Passing threshold
% of bases with a quality score > Q30 (%Q30)	2 × 301 bp: >70% 2 × 251 bp: >75% 2 × 151 bp: >80% 2 × 76 bp: >85%
Cluster passing filter	>75%
PhiX aligned	0.5–10%
PhiX error rate	<6%
Number of bases after trimming (per sample)	>150,000,000
Fraction of closest NCBI reference covered	>0.9
Number of contigs in *de novo* assembly	<300
N50 of the *de novo* assembly	>20,000
Average coverage of the *de novo* assembly	>30
Assembly length	4.5–5.5 Mb

The International Organization for Standardization (ISO) is currently in the process of developing a standard for WGS for typing and genomic characterization of food-borne bacteria^17^. Once publicly available, this ISO standard might contribute to a revision of quality parameters currently in place in different laboratories. Based on the draft assemblies produced by AQUAMIS, the Bakcharak pipeline (see text footnote 10) then conducts a thorough characterization of the *Salmonella* genome. The Bakcharak pipeline provides a detailed characterization for each sample including serotype, 7-gene MLST, resistome, plasmidome and virulome. Since all software tools and databases are standardized within the pipeline, all partners can straightforwardly arrive at comparable characterization results. The characterization results provide important additional information to the phylogeny, as they allow the detailed study of the transmission of particular resistance mechanisms and plasmids.

**FIGURE 3:**
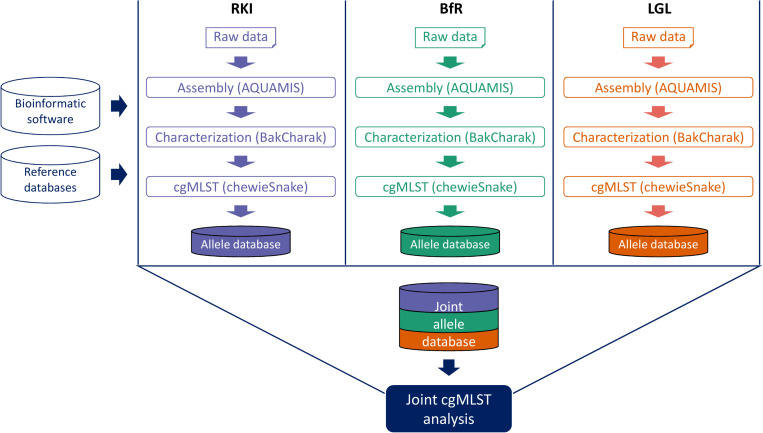
Schematic overview of decentralized pipeline workflow. Standardized bioinformatics software and relevant reference databases are installed at the computing systems of every project partner (BfR, RKI, and LGL). Raw data generated at each partner site runs through the same set of analysis pipelines. Resulting allele profiles are shared and a joint cgMLST analysis is performed.

CgMLST analysis is performed on the draft assemblies with the chewieSnake pipeline (see text footnote 13) by implementing the assembly-based software chewBBACA (Silva et al., 2018). As a central feature, the nucleotide sequence of identified allele variants (including novel alleles) are converted to allele hashes. This ensures that identical allele variants are always assigned the same allele number, irrespective of analysis location and execution. Hence, when all partners analyze sequenced isolates in the same manner, i.e., using the same pipelines, reference databases (e.g., cgMLST scheme) and underlying software programs, the exchange of hashed allele profiles is feasible and sufficient for the joint interpretation of the analysis results. In particular, after exchange of the shared hashed allele profiles a joint allele distance matrix and a common minimum spanning tree can be calculated (Figure 3). Based on the joined allele distance matrix, samples are hierarchically clustered and assigned into cluster groups using a threshold of 10 AD. A unique and stable cluster name is assigned to each cluster group, thus providing a intercommunicable cluster nomenclature. Finally, all clustering information is summarized in a clustering report that is shared between the partners. The minimum spanning tree can be easily visualized together with the characterization results and important metadata information in Grapetree (Zhou et al., 2018).

The exchange of metadata and their unambiguous correlation to sequence data are crucial for a successful evaluation of transmission dynamics. Within the GenoSalmSurv project, the exchange of metadata is limited to isolate source attributes (categorized as shown Table 4), name of the state which forwarded the isolate, isolate submission date, isolate sampling date and information about phenotypical resistance (if available). Metadata information is securely stored throughout the project on our own server infrastructure (hosted at the BfR), physically apart from the cgMLST allele database. Cloud access to the metadata information is password protected and read/write permission is granted to all project partners. New data is added to the interactive table, whenever new allele profiles are exchanged. A consistent isolate naming scheme guarantees an automatic matching of metadata information to nodes of the phylogenetic tree.

**TABLE 4:** Details of minimal isolate source attributes that are exchanged between project partner.

Food^1^	Farm animal^1^	Human^1^	Animal^1^	Environment^1^	Feed^1^
Pork	Laying hen	Blood	Wild animal	Slurry	Feed plant origin
Beef	Broiler	Stool	Domestic animal	Water	Feed animal origin
Poultry meat	Turkey	Urine	Zoo animal	Other	Other
Other/mixed meat	Other poultry	Other	Laboratory animal		
Eggs	Cattle	Unknown	Other		
Other foods animal origin	Pig		Unknown		
Other foods plant origin	Other				
Other	Unknown				
Unknown					

*^1^Isolates which cannot be assigned to one of the listed attributes can be categorized as ‘other’ or ‘unknown.’ A free text field for additional information is available.*

### Interpretation of Cluster Assignment

The prospective monitoring of *Salmonella* spp. using WGS, in which sequenced genomes are continuously added to previously sequenced genomes, can generate actionable cluster information for epidemiologists. However, the formation and the interpretation of WGS clusters must be consistent and robust over time. Differences between genomes are usually calculated by pairwise comparison of the isolates. The resulting matrix contains the number of allele differences between two isolates (Timme et al., 2018). For visualization, a tree is built using a distance-based hierarchical method [e.g., unweighted pair group method with arithmetic mean (UPGMA)] based on cgMLST/whole-genome MLST (wgMLST) analysis or a non-hierarchical method (e.g., maximum-likelihood) based on SNP analysis (Schürch et al., 2018; Uelze et al., 2020). For a first screening, many laboratories apply a specific allele or SNP threshold value for allocation of isolates to a WGS cluster. For *Salmonella*, practical thresholds for interpreting clusters in regulatory applications have been published on SNP counts (Pightling et al., 2018) and cgMLST alleles (Besser et al., 2019). A suggested working definition for *Salmonella* clusters includes ≥3 cases within a 60-day period with 0–10 alleles difference (depending on the applied typing scheme), where at least two cases are related by at most five differences (Besser et al., 2019). However, this liberal allele difference might not prove useful for very clonal serovars, e.g., *S.* Enteritidis, or point source outbreaks. Furthermore, the sole setting of a distance threshold for inclusion or exclusion of isolates to an outbreak should be used with caution and not without considering the epidemiological context (EFSA Panel on Biological Hazards, 2019). Generally, cases with one or more epidemiological links and near-identical pathogen genome sequences, can be supposed to be infected by the same source or vehicle. Individual cases within a WGS cluster for whom no spatio-temporal link could be established are more difficult to allocate to a distinct outbreak event.

The evolution rates of certain *Salmonella* serovars over time have been estimated recently to be approximately 1 to 5 bases per year per genome (Hawkey et al., 2013; Deng et al., 2014; Phillips et al., 2016; Leekitcharoenphon et al., 2019). Such mutation rates are calculated by temporally structured sequence analyses within a Bayesian framework based on sets of isolates giving an indication for the rate of mutational changes within a population over the time (Leekitcharoenphon et al., 2019). Nevertheless, recognizing the dimension of an outbreak, possibly running over years and consisting of several WGS subclusters, requires extensive consideration of epidemiological data and an understanding of the biology of the pathogen itself, as well as the environmental conditions in which the pathogen persists. For example, stress responses to harsh conditions such as chemical substances (e.g., disinfection), temperature, acid, dryness or the host environment lead to prolonged generation time, increased mutability, or reduction of a particular *Salmonella* population (Jayeola et al., 2020). The persistence of *Salmonella* spp. for long periods of time in dry food production environments has been observed (Hoffmann et al., 2020). Further, it is known that *Salmonella* spp. are capable of biofilm formation. Therefore, the biological peculiarities of particular *Salmonella* strains may confound data interpretation during outbreak investigations.

## Discussion

Whole genome sequencing technologies have pushed the development for bacterial genome comparisons and advanced typing approaches. The successful identification of (cross-border) transmission and outbreaks analyses requires the comparative analyses of genome data across sectors and borders. A number of international WGS-based surveillance platforms (Uelze et al., 2020) have been developed up to date, such as Pathogenwatch (see text footnote 1), NCBI pathogen detection (see text footnote 2) and GenomeTrakr (Timme et al., 2018) which utilize either cgMLST or SNP typing approaches to identify food-borne pathogen outbreaks. All three platforms require that raw sequence data is available in a central data storage, followed by a centralized analysis and cluster detection of the genome data. Currently, these platforms represent the most promising solution for a global food-borne disease surveillance strategy. However, many countries and institutions only hesitantly participate in these networks (especially where the sharing of metadata along with genome sequence data is concerned), often due to existing data protection guidelines, regulations, respective research interests or privacy concerns. This results in many missed opportunities for important cluster detection and wastes the potential which is encompassed by the generation of valuable sequence data. Although we strongly encourage the participation in international surveillance networks, we recognize the specific barriers which frequently prevent large-scale, real-time data sharing.

To address this issue, we have developed the GenoSalmSurv project as a working model for an integrated genome-based surveillance of *Salmonella enterica*, which permits reliable cluster identification without sequence data exchange.

Our concept is based on a decentralized analysis of hashed cgMLST allele profiles with open-source bioinformatics tools. Crucially this approach eliminates the need for a large centrally organized and synchronized cgMLST scheme database and sequence database and simplifies the data sharing process, as the exchange of small text files is sufficient for the compilation of data for cluster identification and analysis. In comparison to the aforementioned central analysis platforms, our approach improves scalability and speed of analysis, as time- and resource-consuming data storage, management and processing needs are distributed, and upload and download times are minimized. These are major advantages compared to a central system, which is continuously required to expand, in order to meet the needs of the exponentially growing amount of sequencing data, which places increasingly higher demands for storage and processing of these data on the central computing/storage unit.

Furthermore, as the exchange of data is limited to the essential information necessary for cluster analysis (i.e., the cgMLST allele profiles) our approach provides a practical solution for laboratories which are restricted in their ability to share/exchange sequence data, for example during outbreak situations. An exchange of the raw sequencing data – for instance by uploading to open-access databases – can then be accomplished at a later time point and is highly recommended for in-depth analysis, independent confirmation and integration with international surveillance networks. In addition, the integration of comprehensive and standardized epidemiological data is of utmost importance for reliable data interpretation.

Overall, our objective is to provide accessible, “easy to handle” and standardized tools for the high-resolution method of genome analysis for cluster detection, pathogen monitoring and infection source analysis to all authorities involved in the surveillance of *Salmonella.* Our working model is built around practical solutions for data sharing, with the aim to enable all laboratories to contribute their genomic data to an integrated genome-based surveillance and outbreak analyses with uniform interpretation of results.

During the next project stage, the developed open-source tools will be optimized in regards to user friendliness and backward compatibility and an administration portal to manage user accounts and access rights will be established. The results of a currently ongoing real-time study will be used to validate the established workflow and will further be incorporated into training measures. It is further planned to carry out a benchmarking test with selected public health laboratories already performing WGS, to determine and compare their sequence data quality. Finally, authorities and multipliers will be surveyed, consulted and trained with the aim to make genome-based surveillance and outbreak analyses easily accessible to all authorities involved and thus to accelerate its establishment across all sectors (one-health concept).

## Data Availability Statement

The code for the AQUAMIS, Bakcharak, and chewieSnake bioinformatic pipelines is freely available from GitLab (https://gitlab.com/bfr_bioinformatics/).

## Author Contributions

LU and BM structured the manuscript. LU, NB, MB, UB, AD, CD, JF, AF, SH, IH, UM, JL, MP, SS, AS, SHT, IS, and BM wrote the manuscript. CD and ST developed the in-house bioinformatic pipelines used for analysis of the sequencing data. NB, MP, and CD created the figures. All authors read and approved the manuscript.

## Conflict of Interest

The authors declare that the research was conducted in the absence of any commercial or financial relationships that could be construed as a potential conflict of interest.

## Footnotes

1 https://pathogen.watch/

2 https://www.ncbi.nlm.nih.gov/pathogens/

3 Act on the Prevention and Control of Human Infectious Diseases in Man (IfSG) of 20 July 2002. BGBl. I p. 1045, as amended by article 5 of the Law of 19 June 2020. BGl. I p. 1385. Available at: https://www.gesetze-im-internet.de/ifsg/

4 Food, Feed and Consumer Goods Code in the version of the announcement of 3 June 2013. BGBl. I p. 1426, as amended by article 97 of the Regulation of 19. June 2020. BGBl I p. 1328. Available at: https://www.gesetze-im-internet.de/lfgb/LFGB.pdf

5 Directive 2003/99/EC of the European Parliament and of the Council of 17 November 2003 on the monitoring of zoonoses and zoonotic agents, amending Council Decision 90/424/EEC and repealing Council Directive 92/117/EEC. OJ L 325, 12 December 2003 pp. 31–40, as amended by Council Directive 2013/20/EU of 13 May 2013. OJ L 158 p. 234, 10. June 2013. Available at: https://eur-lex.europa.eu/legal-content/EN/TXT/PDF/?uri=CELEX:02003L0099-20130701&from=EN

6 Regulation (EC) No 2160/2003 of the European Parliament and of the Council of 17 November 2003 on the control of *Salmonella* and other specified food-borne zoonotic agents. OJ L 325, 12 December 20. Available at: https://eur-lex.europa.eu/LexUriServ/LexUriServ.do?uri=OJ:L:2003:325:0001:0015:EN:PDF

7 https://gitlab.com/bfr_bioinformatics/AQUAMIS

8 https://github.com/tseemann/shovill

9 https://github.com/ablab/quast

10 https://gitlab.com/bfr_bioinformatics/bakcharak

11 https://github.com/tseemann/abricate

12 https://github.com/tseemann/mlst

13 https://gitlab.com/bfr_bioinformatics/chewieSnake

14 http://enterobase.warwick.ac.uk/species/index/senterica

15 https://survstat.rki.de/

16 Regulation on the protection against salmonellosis in cattle (cattle salmonellosis regulation) of 14 November 1991. BGBl. p. 2118, amended by article 2 of 17 April 2014. BGBl. p. 388. Available at: https://www.gesetze-im-internet.de/rindsalmv/RindSalmV.pdf

17 ISO/CD 23418. Available at: https://www.iso.org/standard/75509.html

## Abbreviations

BfR: German Federal Institute for Risk Assessment
cgMLST: core genome multilocus sequence typing
EFSA: European Food Safety Authority
EC: European Commission
ECDC: European Centre for Disease Prevention and Control
EU: European Union
FAO: The Food and Agriculture Organization
FLI: Friedrich-Loeffler-Institut
GenoSalmSurv: German Ministry of Health funded project “Integrated genome-based surveillance of *Salmonella*”
IGS: integrated genome-based surveillance
LGL: Bavarian Health and Food Safety Authority
MLST: multilocus sequence typing
MLVA: multiple locus variable-number tandem repeat analysis
NGS: next generation sequencing
NRC-*Salmonella*: National Reference Centre for *Salmonella* and other Bacterial Enteric Pathogens (RKI)
NRL-*Salmonella*: National reference laboratory for *Salmonella* (BfR)
NRL for Salmonellosis in Cattle: National reference laboratory for Salmonellosis in Cattle (FLI)
PCR: polymerase chain reaction
PFGE: pulsed-field gel electrophoresis
QC: quality control
RKI: Robert Koch Institute
*S*.: *Salmonella enterica* subsp. *enterica*
SNP: single nucleotide polymorphism
UPGMA: unweighted pair group method with arithmetic mean
wgMLST: whole genome multilocus sequence typing
WGS: whole genome sequencing.
